# Fractional Herglotz variational problems with Atangana–Baleanu fractional derivatives

**DOI:** 10.1186/s13660-018-1635-9

**Published:** 2018-02-17

**Authors:** Jianke Zhang, Luyang Yin, Chang Zhou

**Affiliations:** Department of Mathematics, School of Science, Xi’an University of Posts and Telecommunications, Xi’an, China

**Keywords:** Fractional variational Herglotz problems, Atangana–Baleanu fractional derivative, Euler–Lagrange equations

## Abstract

The purpose of this paper is to solve fractional calculus of variational Herglotz problem depending on an Atangana–Baleanu fractional derivative. Since the new Atangana–Baleanu fractional derivative is non-singular and non-local, the Euler–Lagrange equations are proposed for the problems of Herglotz. Fractional variational Herglotz problems of variable order are considered and two cases are shown. The Noether-type theorem with this new fractional derivative is proved. Several typical examples of the results of this paper are expressed in this paper.

## Introduction

It is certain for the development of fractional calculus because the fractional derivative has global correlation, which can reflect the historical process of the systematic function. Besides, a fractional derivative model can give better results by using fewer parameters than the classical integer order model. What is more, compared with the nonlinear model, the physical meaning of the fractional order model is clearer and the expression is more concise. Since the first monograph on fractional calculus was published by Oldham and Spanier in 1974, fractional calculus has been widely used in science, engineering, and many other fields [1–4].

With regard to the definition of fractional derivatives, many mathematicians studied fractional derivatives from different aspects and advanced different definitions for them, e.g., Riemann–Liouville (RL) derivative and Caputo derivative. Zhang et al. [5] used the Riemann–Liouville fractional derivative to prove the stability of the linear degenerate fractional differential system. Almeida [6] solved some fractional variational problems involving the Caputo fractional derivative. Nevertheless, both of them have some shortcomings. For example, the Riemann–Liouville derivative cannot be employed without initial conditions, which makes it hard to use in several practical problems. Besides, the fractional calculus defined by Caputo has a singular kernel.

Recently, in 2015, Caputo and Fabrizio [7] proposed Caputo–Fabrizio (CF) fractional derivative to overcome the drawback of the singular kernel. However, since the kernel in the integral is still non-local, the CF fractional derivative also has shortcomings. Moreover, in CF fractional derivatives, the associated integral is not a fractional operator. Herglotz [8] introduced the generalized variational principle in 1930. Then Garra et al. [9], using Caputo derivative calculates, attained Euler–Lagrange equations and transversality conditions for the problem of Herglotz.

In 2016, Atangana and Baleanu [10] introduced a new kind of fractional derivative. Atangana et al. [11] studied the numerical approximation of the Riemann–Liouville derivatives, which is called Atangana–Baleanu (AB) fractional derivative. Several comparative studies of CF fractional derivative and AB fractional derivative have been presented in recent years. Sheikh et al. [12] compared the CF fractional derivative with the AB fractional derivative in the free convection of generalized Casson fluid. The differences between the CF fractional derivative and the AB fractional derivative with respect to the Allen–Cahn model are proposed by Algahtani et al. [13]. The research of Sheikh et al. [14] showed the comparisons of CF fractional derivative with AB fractional derivative in the generalized Casson fluid model, whose exact solutions can be attained by the Laplace transform [15–17]. Khan et al. [18] analyzed the similarities and differences between the two fractional derivatives.

In summary, the fractional derivatives defined in the previous papers have many drawbacks. The derivative of the constant in RL sense does not equal zero. In addition, both RL and Caputo fractional derivatives are singular. Some integral of CF fractional derivative is not a fractional operator. Besides, the generalized Mittag-Leffler function was used by the AB fractional derivative as a non-singular and non-local kernel. What is more, the AB fractional derivative contains all the properties of fractional derivatives. Many researches have tried to use the AB fractional derivative to solve some practical problems. Gómezaguilar et al. [19] utilized the AB fractional operator in dielectric media as an alternative solution of the fractional waves. Alkahtani [20], using the AB fractional derivative, did numerical research on the extension model of dynamics for Chua’s circuit. Gómez-Aguilar et al. [21] attained the analytical solutions for the electrical series circuits RC, LC, and RL with AB fractional derivative. Jan et al. [22] applied the AB fractional derivative into the generalized model of Brinkman-type fluid. Abro et al. [23] solved partial differential equations with AB fractional derivative in molybdenum disulfide nanofluids.

The main aim of this paper is to solve fractional calculus of variational Herglotz problem depending on an AB fractional derivative. The structure of this paper is as follows. In Sect. 2, the basic definitions and notations involving in the Atangana–Baleanu–Riemann–Liouville and Atangana–Baleanu–Caputo fractional derivatives are presented. In Sect. 3, the fractional differential equation of Herglotz is received. In Sect. 4, fractional variational Herglotz problems of variable order are considered. In Sect. 5, the Noether theorem with this new fractional derivative is proved. At last, the conclusion is presented in Sect. 6.

## Preliminaries

In this section, we quote some basic definitions with respect to the Atangana–Baleanu–Riemann–Liouville (ABR) fractional derivative and the Atangana–Baleanu–Caputo (ABC) fractional derivative [24]. Let a function $f(t):[a,b]\rightarrow R$, the left ABR fractional derivative is defined by

1 $$\begin{aligned} {}^{\mathrm {ABR}}_{a}D^{\alpha}_{t}f(t)=\frac{B(\alpha)}{1-\alpha} \frac{d}{dt} \int^{t}_{a}f(x)E_{\alpha} \biggl(-\alpha \frac{(t-x)^{\alpha}}{1-\alpha} \biggr)\,dx, \end{aligned}$$

and in the right ABR fractional derivative sense by

2 $$\begin{aligned} {}^{\mathrm {ABR}}_{t}D^{\alpha}_{b}f(t)=-\frac{B(\alpha)}{1-\alpha} \frac{d}{dt} \int^{b}_{t}f(x)E_{\alpha} \biggl(-\alpha \frac{(x-t)^{\alpha}}{1-\alpha} \biggr)\,dx, \end{aligned}$$

where $B(\alpha)$ is a normalization function such that $B(0) = B(1) = 1$, $0<\alpha<1$, and $E_{\alpha}(z)=\sum^{\infty}_{k=0}\frac {z^{k}}{\Gamma(\alpha k+1)}$ ($z\in C, \operatorname{Re}(\alpha)>0$) is a Mittag-Leffler function with one parameter [25].

### Definition 1

(see [24, Definition 2.1 and Definition 2.5])

Define a Hilbert $\operatorname{space}(H)$, and let a function $f\in H^{1}(a,b)$, $b>a$, $\alpha\in(0,1)$, then the new ABC fractional derivative is defined by

3 $$\begin{aligned} {}^{\mathrm {ABC}}_{a}D^{\alpha}_{t}f(t)=\frac{B(\alpha)}{1-\alpha} \int^{t}_{a}f^{\prime}(x)E_{\alpha} \biggl(-\alpha\frac{(t-x)^{\alpha}}{1-\alpha} \biggr)\,dx \end{aligned}$$

and

4 $$\begin{aligned} {}^{\mathrm {ABC}}_{t}D^{\alpha}_{b}f(t)=-\frac{B(\alpha)}{1-\alpha} \int^{b}_{t}f^{\prime}(x)E_{\alpha} \biggl(-\alpha\frac{(x-t)^{\alpha}}{1-\alpha} \biggr)\,dx. \end{aligned}$$

### Definition 2

(see [24, Proposition 2.4])

Let $g(t)$ be a continuous function and $f(t)$ be of class $C^{1}$. Then we have

5 $$\begin{aligned} \int^{b}_{a}g(t){}^{\mathrm {ABC}}_{a}D^{\alpha}_{t}f(t)\,dt= \int^{b}_{a}f(t){}^{\mathrm {ABR}}_{t}D^{\alpha}_{b}g(t)\,dt+ \biggl[\frac{B(\alpha)}{1-\alpha}f(t)\mathbf{E}^{1}_{\alpha,1,\frac {-\alpha}{1-\alpha},b^{-}}g(t) \biggr]^{t=b}_{t=a} \end{aligned}$$

and

6 $$\begin{aligned} \int^{b}_{a}g(t){}^{\mathrm {ABC}}_{t}D^{\alpha}_{b}f(t)\,dt= \int^{b}_{a}f(t){}^{\mathrm {ABR}}_{a}D^{\alpha}_{t}g(t)\,dt- \biggl[\frac{B(\alpha)}{1-\alpha}f(t)\mathbf{E}^{1}_{\alpha,1,\frac {-\alpha}{1-\alpha},a^{+}}g(t) \biggr]^{t=b}_{t=a}. \end{aligned}$$

Besides, the left generalized fractional integral operator [24] is defined by

7 $$\begin{aligned} \mathbf{E}^{\gamma}_{\rho,\mu,\omega,a^{+}}\varphi(x)= \int^{x}_{a}(x-t)^{\mu-1}E^{\gamma}_{\rho,\mu} \bigl[\omega(x-t)^{\rho} \bigr]\varphi(t)\,dt,\quad x>a, \end{aligned}$$

and the right generalized fractional integral operator is defined by

8 $$\begin{aligned} \mathbf{E}^{\gamma}_{\rho,\mu,\omega,b^{-}}\varphi(x)= \int^{b}_{x}(t-x)^{\mu-1}E^{\gamma}_{\rho,\mu} \bigl[\omega(t-x)^{\rho} \bigr]\varphi(t)\,dt,\quad x< b, \end{aligned}$$

where the generalized Mittag-Leffler function [25] is defined by

$$\begin{aligned} E^{\gamma}_{\rho,\mu}(z)=\sum^{\infty}_{k=0} \frac{(\gamma)_{k}z^{k}}{\Gamma(k\rho+\mu)k!}\quad\bigl(\rho,\mu ,\gamma\in C,\operatorname{Re}(\rho)>0 \bigr). \end{aligned}$$

## Fractional variational Herglotz problems

In this section, we consider fractional variational Herglotz problems with dependence on ABC fractional derivatives and ABR fractional derivatives. According to the generalized variational principle of Herglotz in the shape space [26], the Herglotz problem of the nonconservative system in the phase space can be defined as

9 $$\begin{aligned} \frac{dz}{dt}=L \bigl(t,x(t),{}^{\mathrm {ABC}}_{a}D^{\alpha}_{t}x(t),z(t) \bigr),\quad t\in[a,b], \end{aligned}$$

where the initial condition is $z(a)=z_{a}$, and $z_{a}$ is a given real number. The aim of Herglotz problems is to find a locus $x\in C^{1}([a,b],R)$ with the condition that *z* is the solution of the above system.

The following hypothesis should be allowed:

1. $x(a)=x_{a}$, $x(b)=x_{b}$, $x_{a},x_{b}\in R$;
2. ${}^{\mathrm {ABC}}_{a}D^{\alpha}_{t}x(t)\in C^{1}([a,b], R)$;
3. $L\in C^{1}([a,b]\times R^{3}, R)$;
4. The map $t\mapsto\lambda(t)\partial_{3}L[x,z](t)$ is such that ${}^{\mathrm {ABC}}_{t}D^{\alpha}_{b}(\lambda(t)\partial_{3}L[x,z](t))$ exists and is continuous on $[a,b]$, where
  $$\begin{aligned}{} [x,z](t)= \bigl(t,x(t),{}^{\mathrm {ABC}}_{a}D^{\alpha}_{t}x(t),z(t) \bigr) \end{aligned}$$
  and
  10 $$\begin{aligned} \lambda(t)=\exp\biggl(- \int^{t}_{a}\partial_{4}L[x,z](\tau)\,d\tau\biggr). \end{aligned}$$

### Remark 1

The solution *z* of differential equation (9) lies on the variables *x* and *t*. We consider the function $h(t)\in C^{1}([a,b],R)$ with the boundary conditions $h(a)=h(b)=0$. In addition, we introduce any arbitrary small parameter $\epsilon\in R$, and then $x+\epsilon h\in C^{1}([a,b],R)$ exists in the neighborhood of *x*. Let *x* be replaced by $x+\epsilon h$, then the solution *z* relates to *ϵ* and it is also differentiable with regard to *ϵ*.

Let $z(t)|_{t=b}$ be a minimum and the value of the function $z(x,b)$ be an extremum for the function $x(t)$.

### Theorem 1

*Let* *x* *be such that* $z(x,b)$ *defined by equation* (9) *reaches an extremum*. *Then* *x* *is a solution of*

11 $$\begin{aligned} \lambda(t)\partial_{2}L[x,z](t)+{}^{\mathrm {ABR}}_{t}D^{\alpha}_{b} \bigl(\lambda(t)\partial_{3}L[x,z](t) \bigr)=0 \end{aligned}$$

*on* $[a,b]$.

### Proof

Since $z(x,b)$ is a minimum, with any sufficiently small real number *ϵ*, the solution *z* is given by

12 $$\begin{aligned} \phi(t)=\frac{d}{d\epsilon}z(x+\epsilon h,t)\bigg|_{\epsilon=0}. \end{aligned}$$

Then we attain

13 $$\begin{aligned} \frac{d}{dt}z(x+\epsilon h)=L \bigl(t,x(t)+\epsilon h(t),{}^{\mathrm {ABC}}_{a}D^{\alpha}_{t} \bigl[x(t)+\epsilon h(t) \bigr],z \bigl(x+\epsilon h(t),t \bigr) \bigr). \end{aligned}$$

Therefore, we get

$$\begin{aligned} \frac{d}{dt}\phi(t) =&\frac{d}{dt}\frac{d}{d\epsilon}z(x+\epsilon h)\bigg|_{\epsilon=0} \\ =&\frac{d}{d\epsilon}\frac{d}{dt}z(x+\epsilon h)\bigg|_{\epsilon=0} \\ =&\frac{d}{d\epsilon}L \bigl(t,x(t)+\epsilon h(t),{}^{\mathrm {ABC}}_{a}D^{\alpha}_{t} \bigl[x(t)+\epsilon h(t) \bigr],z \bigl(x+\epsilon h(t),t \bigr) \bigr)\bigg|_{\epsilon=0} \\ =&\partial_{2}L[x,z](t)h(t)+\partial_{3}L[x,z](t){}^{\mathrm {ABC}}_{a}D^{\alpha }_{t}h(t)+ \partial_{4}L[x,z](t)\phi(t). \end{aligned}$$

We denote that

$$\begin{aligned} a(t)=\partial_{4}L[x,z](t) \end{aligned}$$

and

$$\begin{aligned} b(t)=\partial_{2}L[x,z](t)h(t)+\partial _{3}L[x,z](t){}^{\mathrm {ABC}}_{a}D^{\alpha}_{t}h(t). \end{aligned}$$

Then we get

$$\begin{aligned} \frac{d}{dt}\phi(t)=b(t)+a(t)\phi(t), \end{aligned}$$

and considering $\lambda(t)=\exp(-\int^{t}_{a}\partial _{4}L[x,z](\tau)\,d\tau)$, we derive that

$$\begin{aligned} \biggl[\frac{d}{dt}\phi(t)-a(t)\phi(t) \biggr]\lambda(t)=b(t)\lambda (t), \end{aligned}$$

then

$$\begin{aligned} \int_{a}^{t}d \bigl(\phi(\tau)\lambda(\tau) \bigr)= \int_{a}^{t}b(\tau)\lambda(\tau)\,d\tau. \end{aligned}$$

According to the condition that $z(b)$ is minimum, we obtain the solution for the last differential equation:

$$\begin{aligned} \phi(b)\lambda(b)-\phi(a) =& \int_{a}^{b}\lambda(t) \bigl[\partial _{2}L[x,z](t)h(t)+\partial_{3}L[x,z](t){}^{\mathrm {ABC}}_{a}D^{\alpha}_{t}h(t) \bigr]\,dt \\ =& \int_{a}^{b}\lambda(t)\partial _{2}L[x,z](t)h(t)\,dt+ \int_{a}^{b}h(t){}^{\mathrm {ABR}}_{t}D^{\alpha}_{b} \bigl[\lambda(t)\partial_{3}L[x,z](t) \bigr]\,dt \\ & {}+\frac{B(\alpha)}{1-\alpha}h(t)\mathbf{E}^{1}_{\alpha,1,\frac {-\alpha}{1-\alpha},b^{-}} \bigl[\lambda (t)\partial_{3}L[x,z](t) \bigr]\big|^{t=b}_{t=a}. \end{aligned}$$

Since $\phi(a)=\phi(b)=0$ and $h(a)=h(b)=0$, we get

$$\begin{aligned} \lambda(t)\partial_{2}L[x,z](t)+{}^{\mathrm {ABR}}_{t}D^{\alpha}_{b} \bigl(\lambda(t)\partial_{3}L[x,z](t) \bigr)=0 \end{aligned}$$

for all $t\in[a,b]$. □

### Example 1

Consider this system

$$ \textstyle\begin{cases} \frac{dz}{dt}= [{}^{\mathrm {ABC}}_{0}D^{\alpha}_{t}x(t) ]^{2}+t-1,\quad t\in[0,1], \\ x(0)=1,\quad\quad z(0)=0, \end{cases} $$

where $0<\alpha<1$. Since the initial value $x(0)=1$, let $x\equiv1$, and according to Theorem 1, we obtain

$$\begin{aligned} {}^{\mathrm {ABR}}_{t}D^{\alpha}_{1} \bigl[{}^{\mathrm {ABC}}_{0}D^{\alpha}_{t}x(t) \bigr]=0. \end{aligned}$$

## Fractional variational Herglotz problems of variable order

In this section, the fractional operators of variable fractional order with two functions are considered for the Herglotz problems. Then we show two cases: one with one independent variable, another with several independent variables.

In the first part of this section, we discuss the case of one independent variable. Inspired by the paper [27], we introduce a linear combination of the fractional derivatives of variable fractional order by considering the previous concepts.

### Definition 3

(see [27])

Let $\mu,\gamma :[a,b]^{2}\mapsto(0,1)$ be two functions and $\eta=(\eta_{1},\eta _{2})\in[0,1]^{2}$ be a vector. We deduce that the combined ABR fractional derivative is defined by

14 $$\begin{aligned} {}^{\mathrm {ABR}}D^{\mu,\gamma}_{\eta}x(t)=\eta_{1}{{}^{\mathrm {ABR}}_{a}D^{\mu}_{t}}x(t)+ \eta_{2}{{}^{\mathrm {ABR}}_{t}D^{\gamma}_{b}}x(t). \end{aligned}$$

Therefore, the combined ABC fractional derivative is defined by

15 $$\begin{aligned} {}^{\mathrm {ABC}}D^{\mu,\gamma}_{\eta}x(t)=\eta_{1}{{}^{\mathrm {ABC}}_{a}D^{\mu}_{t}}x(t)+ \eta_{2}{{}^{\mathrm {ABC}}_{t}D^{\gamma}_{b}}x(t). \end{aligned}$$

The ABC fractional derivative of variable order is similar to (5) and (6). Then we denote that the dual fractional derivatives of (14) and (15) are

$$\begin{aligned} {}^{\mathrm {ABR}}D^{\gamma,\mu}_{\overline{\eta}}=\eta_{2}{{}^{\mathrm {ABR}}_{a}D^{\gamma}_{t}}+ \eta_{1}{{}^{\mathrm {ABR}}_{t}D^{\mu}_{T}} \end{aligned}$$

and

$$\begin{aligned} {}^{\mathrm {ABC}}D^{\gamma,\mu}_{\overline{\eta}}=\eta_{2}{{}^{\mathrm {ABC}}_{a}D^{\gamma}_{t}}+ \eta_{1}{{}^{\mathrm {ABC}}_{t}D^{\mu}_{T}}, \end{aligned}$$

where $\overline{\eta}=(\eta_{2},\eta_{1})$. Similar to equation (9), the differential equation with a combined ABC fractional derivative of Herglotz is

16 $$\begin{aligned} \frac{dz}{dt}=L \bigl(t,x(t),{}^{\mathrm {ABC}}D^{\mu,\gamma}_{\eta}x(t),z(t) \bigr),\quad t\in[a,b], \end{aligned}$$

and the initial condition is $z(a)=z_{a}$, and $z_{a}$ is a given real number.

We assume that the following conditions:

1. $x(a)=x_{a}$, $x_{a}\in R$;
2. $T\in([a,b], R)$ extremizes the value of $z(T)$;
3. ${}^{\mathrm {ABC}}D^{\mu,\gamma}_{\eta}x(t)\in C^{1}([a,b], R)$;
4. $L\in C^{1}([a,b]\times R^{3}, R)$;
5. The map $t\mapsto\lambda(t)\partial_{3}L[x,z]^{\mu,\gamma }_{\eta}(t)$ is such that ${}^{\mathrm {ABR}}_{T}D^{\gamma}_{t}(\lambda (t)\partial_{3}L[x,z]^{\mu,\gamma}_{\eta}(t))$, ${}^{\mathrm {ABR}}_{a}D^{\gamma}_{t}(\lambda(t)\partial_{3}L[x,z]^{\mu,\gamma }_{\eta}(t))$, and ${}^{\mathrm {ABR}}D^{\gamma,\mu}_{\overline{\eta}}(\lambda (t)\partial_{3}L[x,z]^{\mu,\gamma}_{\eta}(t))$ exist and are continuous on $[a,b]$, where
  $$\begin{aligned}{} [x,z]^{\mu,\gamma}_{\eta}(t)= \bigl(t,x(t),{}^{\mathrm {ABC}}D^{\mu,\gamma }_{\eta}x(t),z(t) \bigr) \end{aligned}$$
  and
  17 $$\begin{aligned} \lambda(t)=\exp\biggl(- \int^{t}_{a}\partial_{4}L[x,z]^{\mu,\gamma}_{\eta}( \tau)\,d\tau\biggr) \end{aligned}$$
  are valid.

### Theorem 2

*Let* *x* *be such that* *z* *defined by equation* (16) *gains an extremum*. *Then* *x* *is a solution of*

18 $$\begin{aligned} \lambda(t)\partial_{2}L[x,z]^{\mu,\gamma}_{\eta }(t)+{}^{\mathrm {ABR}}D^{\gamma,\mu}_{\overline{\eta}} \bigl(\lambda(t)\partial_{3}L[x,z]^{\mu,\gamma}_{\eta}(t) \bigr)=0 \end{aligned}$$

*on* $[a,T]$, *and*

19 $$\begin{aligned} \eta_{2} \bigl({}^{\mathrm {ABR}}_{a}D^{\gamma}_{t} \bigl(\lambda(t)\partial_{3}L[x,z]^{\mu,\gamma}_{\eta}(t) \bigr)-{}^{\mathrm {ABR}}_{T}D^{\gamma}_{t} \bigl(\lambda (t)\partial_{3}L[x,z]^{\mu,\gamma}_{\eta}(t) \bigr) \bigr)=0 \end{aligned}$$

*on* $[T,b]$, *where* $\overline{\eta}=(\eta_{2},\eta_{1})$. *In addition*, *the following condition is satisfied*:

20 $$\begin{aligned} \begin{aligned}[b] & \biggl[\eta_{1}\frac{B(\mu)}{1-\mu}\mathbf{E}^{1}_{\mu,1,\frac {-\mu}{1-\mu},T^{-}} \bigl(\lambda(t)\partial_{3}L[x,z]^{\mu,\gamma}_{\eta}(t) \bigr) \\ &\quad{} -\eta_{2}\frac{B(\gamma)}{1-\gamma}\mathbf{E}^{1}_{\gamma,1,\frac {-\gamma}{1-\gamma},T^{+}} \bigl(\lambda(t)\partial_{3}L[x,z]^{\mu,\gamma}_{\eta}(t) \bigr) \biggr]_{t=T}=0. \end{aligned} \end{aligned}$$

*If* $T< b$, *then* $L[x,z]^{\mu,\gamma}_{\eta}(T)=0$.

### Proof

Let *x* be a solution to the problem and consider an admissible variation of *x*, with any sufficiently small real number *ϵ*. The solution *z* is given by

21 $$\begin{aligned} \theta(t)=\frac{d}{d\epsilon}z(x+\epsilon h,t)\bigg|_{\epsilon=0}, \end{aligned}$$

where $\theta(T)=0$, because $z(T)$ is an extremum.

Then we get

$$\begin{aligned} \frac{d}{dt}\theta(t) =&\frac{d}{dt}\frac{d}{d\epsilon}z(x+\epsilon h)\bigg|_{\epsilon=0} \\ =&\frac{d}{d\epsilon}\frac{d}{dt}z(x+\epsilon h)\bigg|_{\epsilon=0} \\ =&\frac{d}{d\epsilon}L \bigl(t,x(t)+\epsilon h(t),{}^{\mathrm {ABC}}D^{\mu ,\gamma}_{\eta} \bigl[x(t)+\epsilon h(t) \bigr],z \bigl(x+\epsilon h(t),t \bigr) \bigr)\big|_{\epsilon=0} \\ =&\partial_{2}L[x,z]^{\mu,\gamma}_{\eta}(t)h(t)+\partial _{3}L[x,z]^{\mu,\gamma}_{\eta}(t){}^{\mathrm {ABC}}D^{\mu,\gamma}_{\eta}h(t)+ \partial_{4}L[x,z]^{\mu,\gamma}_{\eta}(t)\theta(t). \end{aligned}$$

Considering $\lambda(t)=\exp(-\int^{t}_{a}\partial_{4}L[x,z]^{\mu ,\gamma}_{\eta}(\tau)\,d\tau)$, we get the solution for the differential equation

$$\begin{aligned} \theta(T)\lambda(T)-\theta(a)= \int_{a}^{T}\lambda(t) \bigl[\partial _{2}L[x,z]^{\mu,\gamma}_{\eta}(t)h(t)+\partial _{3}L[x,z]^{\mu,\gamma}_{\eta}(t){}^{\mathrm {ABC}}D^{\mu,\gamma}_{\eta}h(t) \bigr]\,dt. \end{aligned}$$

Since $\theta(a)=0$ and $z(T)$ is an extremum for *x*, $\theta(T)=0$ holds. Then we obtain

22 $$\begin{aligned} \int_{a}^{T}\lambda(t) \bigl[\partial _{2}L[x,z]^{\mu,\gamma}_{\eta}(t)h(t)+\partial _{3}L[x,z]^{\mu,\gamma}_{\eta}(t){}^{\mathrm {ABC}}D^{\mu,\gamma}_{\eta}h(t) \bigr]\,dt=0. \end{aligned}$$

Firstly, we consider the second part in (22) and the definition of combined ABC derivative. We get

$$\begin{aligned}& \int_{a}^{T}\lambda(t)\partial _{3}L[x,z]^{\mu,\gamma}_{\eta}(t){}^{\mathrm {ABC}}D^{\mu,\gamma}_{\eta}h(t)\,dt \\& \quad = \int_{a}^{T}\lambda(t)\partial _{3}L[x,z]^{\mu,\gamma}_{\eta}(t) \bigl(\eta _{1}{{}^{\mathrm {ABC}}_{a}D^{\mu}_{t}}h(t)+ \eta_{2}{{}^{\mathrm {ABC}}_{t}D^{\gamma}_{b}}h(t) \bigr)\,dt \\& \quad =\eta_{1} \int_{a}^{T}\lambda(t)\partial _{3}L[x,z]^{\mu,\gamma}_{\eta}(t) {}^{\mathrm {ABC}}_{a}D^{\mu}_{t}h(t)\,dt \\& \quad\quad{} +\eta_{2} \biggl[ \int_{a}^{b}\lambda(t)\partial _{3}L[x,z]^{\mu,\gamma}_{\eta}(t){}^{\mathrm {ABC}}_{t}D^{\gamma}_{b}h(t))\,dt- \int_{T}^{b}\lambda(t)\partial _{3}L[x,z]^{\mu,\gamma}_{\eta}(t){}^{\mathrm {ABC}}_{t}D^{\gamma}_{b}h(t))\,dt \biggr]. \end{aligned}$$

Considering $\overline{\eta}=(\eta_{2},\eta_{1})$ with Definition 2, and since $h(a)=h(b)=0$, we derive that

$$\begin{aligned}& \int_{a}^{T}\lambda(t)\partial _{3}L[x,z]^{\mu,\gamma}_{\eta}(t){}^{\mathrm {ABC}}D^{\mu,\gamma}_{\eta}h(t)\,dt \\& \quad = \int_{a}^{T}h(t){}^{\mathrm {ABR}}D^{\gamma,\mu}_{\overline{\eta}} \bigl(\lambda(t)\partial_{3}L[x,z]^{\mu,\gamma}_{\eta}(t) \bigr)\,dt \\& \quad\quad{} + \int_{T}^{b}\eta_{2}h(t) \bigl[{}^{\mathrm {ABR}}_{a}D^{\gamma}_{t} \bigl(\lambda (t)\partial_{3}L[x,z]^{\mu,\gamma}_{\eta}(t) \bigr)-{}^{\mathrm {ABR}}_{T}D^{\gamma}_{t} \bigl(\lambda (t)\partial_{3}L[x,z]^{\mu,\gamma}_{\eta}(t) \bigr) \bigr]\,dt \\& \quad\quad{} +h(T) \biggl[\eta_{1}\frac{B(\mu)}{1-\mu}\mathbf{E}^{1}_{\mu ,1,\frac{-\mu}{1-\mu},T^{-}} \bigl(\lambda(t)\partial_{3}L[x,z]^{\mu,\gamma}_{\eta}(t) \bigr) \\& \quad\quad{} -\eta_{2}\frac{B(\gamma)}{1-\gamma}\mathbf{E}^{1}_{\gamma,1,\frac {-\gamma}{1-\gamma},T^{+}} \bigl(\lambda(t)\partial_{3}L[x,z]^{\mu,\gamma}_{\eta}(t) \bigr) \biggr]_{t=T}. \end{aligned}$$

Secondly, from equation (22), we attain the following expression:

$$\begin{aligned}& \int_{a}^{T}h(t) \bigl[\partial _{2}L[x,z]^{\mu,\gamma}_{\eta}(t)\lambda (t)+{}^{\mathrm {ABR}}D^{\gamma,\mu}_{\overline{\eta}} \bigl(\lambda(t)\partial _{3}L[x,z]^{\mu,\gamma}_{\eta}(t) \bigr) \bigr]\,dt \\& \quad{} + \int_{T}^{b}\eta_{2}h(t) \bigl[{}^{\mathrm {ABR}}_{a}D^{\gamma}_{t} \bigl(\lambda (t)\partial_{3}L[x,z]^{\mu,\gamma}_{\eta}(t) \bigr)-{}^{\mathrm {ABR}}_{T}D^{\gamma}_{t} \bigl(\lambda (t)\partial_{3}L[x,z]^{\mu,\gamma}_{\eta}(t) \bigr) \bigr]\,dt \\& \quad{} +h(T) \biggl[\eta_{1}\frac{B(\mu)}{1-\mu}\mathbf{E}^{1}_{\mu ,1,\frac{-\mu}{1-\mu},T^{-}} \bigl(\lambda(t)\partial_{3}L[x,z]^{\mu,\gamma}_{\eta}(t) \bigr) \\& \quad{} -\eta_{2}\frac{B(\gamma)}{1-\gamma}\mathbf{E}^{1}_{\gamma,1,\frac {-\gamma}{1-\gamma},T^{+}} \bigl(\lambda(t)\partial_{3}L[x,z]^{\mu,\gamma}_{\eta}(t) \bigr) \biggr]_{t=T}=0. \end{aligned}$$

Then we obtain the Euler–Lagrange equations (18)–(19) and the transversality conditions (20).

In the second part of this section, we generalize the fractional variational principle of Herglotz to the one involving several independent variables. Define the vector $x=(x_{1},x_{2},\ldots ,x_{n})\in\Omega= \prod^{n}_{i=1}[a_{i},b_{i}]$ with $n\in N$. Consider $u(t,x)$ such that give an extremum to $z[x,T]$, where *z* satisfies the differential equation

23 $$\begin{aligned} \begin{aligned}[b] \frac{dz}{dt}&= \int_{\Omega}L \bigl(t,x,u(t,x),{}^{\mathrm {ABC}}D^{\mu,\gamma}_{\eta} u(t,x), \\ &\quad{} {}^{\mathrm {ABC}}D^{\mu_{1},\gamma_{1}}_{\eta_{1}} u(t,x),\ldots ,{}^{\mathrm {ABC}}D^{\mu_{n},\gamma_{n}}_{\eta_{n}} u(t,x),z(t) \bigr)\,d^{n}x,\quad t \in[a,b], \end{aligned} \end{aligned}$$

where $d^{n}x=dx_{1}\,dx_{2}\,\cdots \,d_{n}$, and assume that

1. $P=[a,b]\times\Omega$, $u(t,x)=g(t,x)$ for all $(t,x)\in \partial P$, where *∂P* is the boundary of *P*, and $g:\partial P\rightarrow R$ is a given function;
2. $\mu, \mu_{i}, \gamma, \gamma_{i}\in(0,1)$ and $\eta, \eta^{1},\ldots, \eta^{n}\in[0,1]^{2}$ with $i=1, 2,\ldots, n$;
3. $L:P\times R^{n+3}\rightarrow R$ is of class $C^{1}$;
4. $(t,x)\mapsto\lambda(t)\partial_{n+2}L[u,z]^{\mu,\gamma }_{n,\eta}(t,x)$ and $(t,x)\mapsto\lambda(t)\partial _{n+3}L[u,z]^{\mu,\gamma}_{n,\eta}(t,x)$ are such that ${}^{\mathrm {ABC}}D^{\mu ,\gamma}_{\eta} u(t,x)$, ${}^{\mathrm {ABC}}D^{\mu_{1},\gamma_{1}}_{\eta_{1}} u(t,x)$, ${}^{\mathrm {ABC}}D^{\mu_{n},\gamma_{n}}_{\eta_{n}} u(t,x)$ exist and are continuous, where
  $$\begin{aligned}{} [u,z]^{\mu,\gamma}_{n,\eta}(t,x) =& \bigl(t,x,u(t,x),{}^{\mathrm {ABC}}D^{\mu ,\gamma}_{\eta} u(t,x), \\ & {}^{\mathrm {ABC}}D^{\mu_{1},\gamma_{1}}_{\eta_{1}} u(t,x),\ldots ,{}^{\mathrm {ABC}}D^{\mu_{n},\gamma_{n}}_{\eta_{n}} u(t,x),z(t) \bigr), \end{aligned}$$
  and
  $$\begin{aligned} \lambda(t)=\exp\biggl(- \int^{t}_{a} \int_{\Omega}\partial_{2n+4}L[u,z]^{\mu,\gamma}_{n,\eta}( \tau,x)\,d^{n}x\,d\tau\biggr). \end{aligned}$$

 □

### Theorem 3

*If* $(u,z)$ *attains an extremum of equation* (23), *then* $(u,z)$ *is a solution of the system of equations*

24 $$\begin{aligned} \begin{aligned}[b] & \lambda(t)\partial_{n+2}L[u,z]^{\mu,\gamma}_{n,\eta }(t,x)+{}^{\mathrm {ABR}}D^{\gamma,\mu}_{\overline{\eta}} \bigl(\lambda(t)\partial_{n+3}L[u,z]^{\mu,\gamma}_{n,\eta}(t,x) \bigr) \\ &\quad{} +\sum^{n}_{i=1}{}^{\mathrm {ABR}}D^{\gamma_{i},\mu_{i}}_{\overline{\eta}^{i}} \bigl(\lambda(t)\partial_{n+3+i}L[u,z]^{\mu,\gamma}_{n,\eta}(t,x) \bigr)=0 \end{aligned} \end{aligned}$$

*on* $[a,T]\times\Omega$, *and*

25 $$\begin{aligned} \eta_{2} \bigl({}^{\mathrm {ABR}}_{a}D^{\gamma}_{t} \bigl(\lambda(t)\partial_{n+3}L[u,z]^{\mu,\gamma}_{n,\eta}(t,x) \bigr)-{}^{\mathrm {ABR}}_{T}D^{\gamma}_{t} \bigl(\lambda (t)\partial_{n+3}L[u,z]^{\mu,\gamma}_{n,\eta}(t,x) \bigr) \bigr)=0 \end{aligned}$$

*on* $[T,b]\times\Omega$, *where* $\overline{\eta}=(\eta_{2},\eta _{1})$ *and* $\overline{\eta}^{i}=(\eta_{2}^{i},\eta_{1}^{i})$. *What is more*, *the following condition is satisfied*:

26 $$\begin{aligned} \begin{aligned}[b] & \biggl[\eta_{1}\frac{B(\mu)}{1-\mu}\mathbf{E}^{1}_{\mu,1,\frac {-\mu}{1-\mu},T^{-}} \bigl(\lambda(t)\partial_{n+3}L[u,z]^{\mu,\gamma}_{n,\eta}(t,x) \bigr) \\ &\quad{} -\eta_{2}\frac{B(\gamma)}{1-\gamma}\mathbf{E}^{1}_{\gamma,1,\frac {-\gamma}{1-\gamma},T^{+}} \bigl(\lambda(t)\partial_{n+3}L[u,z]^{\mu,\gamma}_{n,\eta}(t,x) \bigr) \biggr]_{t=T}=0. \end{aligned} \end{aligned}$$

*If* $T< b$, *then* $L[u,z]^{\mu,\gamma}_{n,\eta}(T,x)=0$.

### Proof

Similar to the proof of Theorem 2, an admissible variation of *u* is considered. We replace $u(t,x)$ with $u(t,x)+\epsilon h(t,x)$, where *ϵ* is any sufficiently small real number. Then the solution *z* is given by

27 $$\begin{aligned} \theta(t)=\frac{d}{d\epsilon}z(u+\epsilon h,t)\bigg|_{\epsilon=0}, \end{aligned}$$

where $\theta(T)=0$, because $z(T)$ is an extremum. Then we obtain

$$\begin{aligned} \frac{d}{dt}\theta(t) =&\frac{d}{dt}\frac{d}{d\epsilon}z(u+\epsilon h)\bigg|_{\epsilon=0} \\ =&\frac{d}{d\epsilon}\frac{d}{dt}z(u+\epsilon h)\bigg|_{\epsilon=0} \\ =&\frac{d}{d\epsilon} \int_{\Omega}L[u+\epsilon h,z]^{\mu,\gamma}_{n,\eta}(t,x)\, d^{n}x\bigg|_{\epsilon=0}. \end{aligned}$$

We conclude that

$$\begin{aligned} \frac{d}{dt}\theta(t) =& \int_{\Omega} \Biggl(\partial_{n+2}L[u,z]^{\mu,\gamma}_{n,\eta }(t,x)h(t,x)+ \partial_{n+3}L[u,z]^{\mu,\gamma}_{n,\eta}(t,x){}^{\mathrm {ABC}}D^{\mu,\gamma }_{\eta}h(t,x) \\ &{}+\sum_{i=1}^{n}\partial _{n+3+i}L[u,z]^{\mu,\gamma}_{n,\eta}(t,x){}^{\mathrm {ABC}}D^{\mu_{i},\gamma _{i}}_{\eta^{i}}h(t,x)+ \partial_{2n+4}L[u,z]^{\mu,\gamma}_{n,\eta}(t,x)\theta(t) \Biggr) \,d^{n}x. \end{aligned}$$

Then we define

$$\begin{aligned} A(t)= \int_{\Omega}\partial_{2n+4}L[u,z]^{\mu,\gamma}_{n,\eta}(t,x)\,d^{n}x, \end{aligned}$$

and

$$\begin{aligned} B(t) =& \int_{\Omega} \Biggl(\partial_{n+2}L[u,z]^{\mu,\gamma}_{n,\eta }(t,x)h(t,x)+ \partial_{n+3}L[u,z]^{\mu,\gamma}_{n,\eta}(t,x){}^{\mathrm {ABC}}D^{\mu,\gamma }_{\eta}h(t,x) \\ &{} +\sum_{i=1}^{n}\partial _{n+3+i}L[u,z]^{\mu,\gamma}_{n,\eta}(t,x){}^{\mathrm {ABC}}D^{\mu_{i},\gamma _{i}}_{\eta^{i}}h(t,x) \Biggr)\,d^{n}x. \end{aligned}$$

Considering $\lambda(t)=\exp(-\int^{t}_{a}\int_{\Omega}\partial _{2n+4}L[u,z]^{\mu,\gamma}_{n,\eta}(\tau,x)\,d^{n}x\,d\tau)$, we attain the solution for the differential equation:

$$\begin{aligned} \theta(T)\lambda(T)-\theta(a)= \int_{a}^{T}B(t)\lambda(t)\,dt. \end{aligned}$$

Since $\theta(a)=0$ and $z(T)$ is an extremum, $\theta(T)=0$ holds. Then we obtain

28 $$\begin{aligned} \int_{a}^{T}B(t)\lambda(t)\,dt=0. \end{aligned}$$

We first consider only the second part in (28), then we get

$$\begin{aligned}& \int_{a}^{T} \int_{\Omega}\partial_{n+3}L[u,z]^{\mu,\gamma}_{n,\eta }(t,x){}^{\mathrm {ABC}}D^{\mu,\gamma}_{\eta}h(t,x)\,d^{n}x \\& \quad = \int_{a}^{T} \int_{\Omega}\lambda(t)\partial_{n+3}L[u,z]^{\mu,\gamma}_{n,\eta}(t,x) \bigl(\eta_{1}{{}^{\mathrm {ABC}}_{a}D^{\mu}_{t}}h(t,x)+ \eta_{2}{{}^{\mathrm {ABC}}_{t}D^{\gamma}_{b}}h(t,x) \bigr)\,d^{n}x\,dt \\& \quad =\eta_{1} \int_{a}^{T} \int_{\Omega}\lambda(t)\partial_{n+3}L[u,z]^{\mu,\gamma}_{n,\eta }(t,x){}^{\mathrm {ABC}}_{a}D^{\mu}_{t}h(t,x)\,d^{n}x\,dt \\& \quad\quad{} +\eta_{2} \biggl[ \int_{a}^{b}\lambda(t)\partial _{n+3}L[u,z]^{\mu,\gamma}_{n,\eta}(t,x){}^{\mathrm {ABC}}_{t}D^{\gamma }_{b}h(t,x))\,d^{n}x\,dt \\& \quad\quad{} - \int_{T}^{b} \int_{\Omega}\lambda(t)\partial_{n+3}L[u,z]^{\mu,\gamma}_{n,\eta }(t,x){}^{\mathrm {ABC}}_{t}D^{\gamma}_{b}h(t,x))\,d^{n}x \,dt \biggr]. \end{aligned}$$

Besides, let $\overline{\eta}=(\eta_{2},\eta_{1})$ and $h(a,x)=h(b,x)=0$ for all $x\in\Omega$, we obtain

$$\begin{aligned}& \int_{a}^{T} \int_{\Omega}\partial_{n+3}L[u,z]^{\mu,\gamma}_{n,\eta }(t,x){}^{\mathrm {ABC}}D^{\mu,\gamma}_{\eta}h(t,x)\,d^{n}x \\& \quad = \int_{a}^{T} \int_{\Omega}h(t,x){}^{\mathrm {ABR}}D^{\gamma,\mu}_{\overline{\eta}} \bigl(\lambda(t)\partial_{n+3}L[u,z]^{\mu,\gamma}_{n,\eta}(t,x) \bigr)\,d^{n}x\,dt \\& \quad\quad{} + \int_{T}^{b}\eta_{2}h(t,x) \bigl[{}^{\mathrm {ABR}}_{a}D^{\gamma}_{t} \bigl(\lambda (t)\partial_{n+3}L[u,z]^{\mu,\gamma}_{n,\eta}(t,x) \bigr) \\& \quad\quad{} -{}^{\mathrm {ABR}}_{T}D^{\gamma}_{t} \bigl(\lambda (t) \partial_{n+3}L[u,z]^{\mu,\gamma}_{n,\eta}(t,x) \bigr) \bigr]\,d^{n}x\,dt \\& \quad\quad{} + \int_{\Omega}h(T,x) \biggl[\eta_{1}\frac{B(\mu)}{1-\mu} \mathbf{E}^{1}_{\mu,1,\frac{-\mu}{1-\mu},T^{-}} \bigl(\lambda (t)\partial _{n+3}L[u,z]^{\mu,\gamma}_{n,\eta}(t,x) \bigr) \\& \quad\quad{} -\eta_{2}\frac{B(\gamma)}{1-\gamma}\mathbf{E}^{1}_{\gamma,1,\frac {-\gamma}{1-\gamma},T^{+}} \bigl(\lambda(t)\partial_{n+3}L[u,z]^{\mu,\gamma}_{n,\eta}(t,x) \bigr) \biggr]_{t=T}. \end{aligned}$$

Similarly, let $\overline{\eta}^{i}=(\eta_{2}^{i},\eta_{1}^{i})$ and $h(a_{i})=h(b_{i})=0$, we get

$$\begin{aligned}& \int^{T}_{a} \int_{\Omega}\lambda(t)\partial_{n+3+i}L[u,z]^{\mu,\gamma }_{n,\eta}(t,x) \bigl(\eta^{i}_{1}{}^{\mathrm {ABC}}_{a_{i}}D^{\mu^{i}}_{x_{i}}h(t,x)+ \eta^{i}_{2}{}^{\mathrm {ABC}}_{x_{i}}D^{\gamma^{i}}_{b_{i}}h(t,x) \bigr)\,d^{n}x\,dt \\& \quad = \int^{T}_{a} \int_{\Omega}h(t,x){}^{\mathrm {ABR}}D_{\overline{\eta}^{i}}^{\gamma_{i},\mu_{i}} \bigl(\lambda(t)\partial_{n+3+i}L[u,z]^{\mu,\gamma}_{n,\eta}(t,x) \bigr)\,d^{n}x\,dt. \end{aligned}$$

Substituting these relations into (28), we deduce that

$$\begin{aligned}& \int_{a}^{T} \int_{\Omega}h(t,x) \Biggl[\partial_{n+2}L[u,z]^{\mu,\gamma }_{n,\eta}(t,x) \lambda(t)+{}^{\mathrm {ABR}}D^{\gamma,\mu}_{\overline{\eta}} \bigl(\lambda(t) \partial_{n+3}L[u,z]^{\mu,\gamma}_{n,\eta}(t,x) \bigr) \\& \quad{} +\sum^{n}_{i=1}{}^{\mathrm {ABR}}D_{\overline{\eta}^{i}}^{\gamma_{i},\mu_{i}} \bigl(\lambda(t)\partial_{n+3+i}L[u,z]^{\mu,\gamma}_{n,\eta}(t,x) \bigr) \Biggr]\,d^{n}x\,dt \\& \quad{} + \int_{T}^{b}\eta_{2}h(t,x) \bigl[{}^{\mathrm {ABR}}_{a}D^{\gamma}_{t} \bigl(\lambda (t)\partial_{n+3}L[u,z]^{\mu,\gamma}_{n,\eta}(t,x) \bigr) \\& \quad{}-{}^{\mathrm {ABR}}_{T}D^{\gamma}_{t} \bigl(\lambda (t) \partial_{n+3}L[u,z]^{\mu,\gamma}_{n,\eta}(t,x) \bigr) \bigr]\,d^{n}x\,dt \\& \quad{}+ \int_{\Omega}h(T,x) \biggl[\eta_{1}\frac{B(\mu)}{1-\mu} \mathbf{E}^{1}_{\mu,1,\frac{-\mu}{1-\mu},T^{-}} \bigl(\lambda (t)\partial _{n+3}L[u,z]^{\mu,\gamma}_{n,\eta}(t,x) \bigr) \\& \quad{}-\eta_{2}\frac{B(\gamma)}{1-\gamma}\mathbf{E}^{1}_{\gamma,1,\frac {-\gamma}{1-\gamma},T^{+}} \bigl(\lambda(t)\partial_{n+3}L[u,z]^{\mu,\gamma}_{n,\eta}(t,x) \bigr) \biggr]_{t=T}=0. \end{aligned}$$

So we get the Euler–Lagrange equations (24)–(25) and the transversality condition (26) with appropriate choices of *h*. □

### Example 2

Consider the system

$$ \textstyle\begin{cases} \frac{dz}{dt}= [{}^{\mathrm {ABC}}D^{\mu,\gamma}_{\eta}x(t) ]^{2}+t^{2}-1,\quad t\in[0,3], \\ x(0)=1,\quad\quad z(0)=0, \end{cases} $$

where $0<\alpha<1$ and $0<\beta<1$. Since the initial condition $x(0)=1$, we attain $x\equiv1$. According to equation (18)–(19) in Theorem 2, we get

$$\begin{aligned} {}^{\mathrm {ABR}}D^{\gamma,\mu}_{\overline{\eta}} \bigl[{}^{\mathrm {ABC}}D^{\mu,\gamma }_{\eta}x(t) \bigr]=0. \end{aligned}$$

Then we obtain

$$ \textstyle\begin{cases} \frac{dz}{dt}=t^{2}-1,\quad t\in[0,3], \\ z(0)=0, \end{cases} $$

whose solution is

$$\begin{aligned} z(t)=\frac{1}{3}t^{3}-t. \end{aligned}$$

Besides, from equation (20), since $L[x,z]^{\mu,\gamma}_{\eta }(T)=0$, we deduce

$$\begin{aligned} T^{2}-1=0, \end{aligned}$$

whose solution is

$$\begin{aligned} T=1, \end{aligned}$$

and the minimum of this system is

$$\begin{aligned} z(1)=-\frac{2}{3}. \end{aligned}$$

### Example 3

Consider the following system:

$$ \textstyle\begin{cases} \frac{dz}{dt}= [x^{3}(t)+z(t)+1 ](t-2),\quad t\in[0,3], \\ x(0)=0,\quad\quad z(0)=0. \end{cases} $$

Let $x\equiv0$ with the initial condition $x(0)=0$, and as is shown in Theorem 2, we attain

$$\begin{aligned} (t-2)x^{2}(t)=0,\quad\forall t\in[0,T]. \end{aligned}$$

Then we get

$$ \textstyle\begin{cases} \frac{dz}{dt}=(t-2) (z(t)+1 ),\quad t\in[0,3], \\ z(0)=0, \end{cases} $$

whose solution is

$$\begin{aligned} z(t)=\exp\biggl(\frac{1}{2}t^{2}-2t \biggr)-1. \end{aligned}$$

Moreover, since $L[x,z]^{\mu,\gamma}_{\eta}(T)=0$, we deduce

$$\begin{aligned} (T-2) \bigl(z(T)+1 \bigr)=0, \end{aligned}$$

whose solution is

$$\begin{aligned} T=2, \end{aligned}$$

and the minimum value of $z(t)$ is

$$\begin{aligned} z(2)=\exp(-2)-1. \end{aligned}$$

## Noether-type theorem

A Noether-type theorem, saying popularly, is symmetric principles. It expresses the correspondence between successive symmetries and conservation laws, the solutions of the Euler–Lagrange equation. In this section, we extend a Noether-type theorem to the one that holds for fractional variational Herglotz-type problems with one (Theorem 4).

Let us discuss the condition with one variable. Consider the one-parameter group of invertible transformations [28]

29 $$\begin{aligned} \overline{x}_{j}=h_{j}(t,x,s),\quad j=1,2,\ldots,n, \end{aligned}$$

where $s\in(-\varepsilon,\varepsilon)$ is a parameter, and $h_{j}$ are of class $C^{2}$ such that $h_{j}(t,x,0)=x_{j}$ for all $j\in {1,2,\ldots,n}$ and for all $(t,x)\in[a,b]\times R^{2}$. From Taylor’s formula, we get

30 $$\begin{aligned} h_{j}(t,x,s)=h_{j}(t,x,0)+s\xi_{j}(t,x)+o(s)=x_{j}+s \xi_{j}(t,x)+o(s), \end{aligned}$$

where $\xi_{j}(t,x)=\frac{\partial h_{j}(t,x,s)}{\partial s}|_{s=0}$. Then we can see $\overline{x}_{j}\approx x_{j}+s\xi_{j}(t,x)$ by the linear approximation to transformation.

By $\psi=\psi(t)$, the total variation of the functional $z=z(x,t)$, which is produced by the family of transformations (29), is given by

31 $$\begin{aligned} \psi(t)=\frac{d}{d s}z(x+\xi s,t)\bigg|_{s=0}. \end{aligned}$$

### Remark 2

The functional *z*, defined by the differential equation (9), is invariant if $\psi(t)\equiv0$.

### Remark 3

When $s=0$, $\overline{z}(\overline {x},t)=z(x,t)$ holds.

### Remark 4

Define the symbol $\mathcal{D}$ as

$$\begin{aligned} \mathcal{D}^{\alpha}[f,g]=f{}^{\mathrm {ABC}}_{a}D^{\alpha}_{t} \cdot g-g\cdot{}^{\mathrm {ABR}}_{t}D^{\alpha}_{b} f. \end{aligned}$$

### Theorem 4

*Let the functional* *z*, *which is defined by differential equation* (9), *be invariant*, *then we have*

32 $$\begin{aligned} \sum_{j=1}^{n}\mathcal{D}^{\alpha_{j}} \biggl[\lambda(t)\frac{\partial L[x,z](t)}{\partial {}^{\mathrm {ABC}}_{a}D^{\alpha_{j}}_{t}x_{j}},\xi_{j}(t,x) \biggr]=0, \end{aligned}$$

*where* $\lambda(t)=\exp(-\int_{a}^{t}\frac{\partial L[x,z](\tau )}{\partial z(x,\tau)}\,d\tau)$ *holds along the solutions of the generalized fractional Euler–Lagrange equations* (3).

### Proof

According to equations (9) and (30), we have

33 $$\begin{aligned} \frac{d\overline{z}}{dt}=L \bigl(t,\overline{x}(t),{}^{\mathrm {ABC}}_{a}D^{\alpha}_{t} \overline{x}(t),\overline{z}(t) \bigr),\quad t\in[a,b]. \end{aligned}$$

Let $s=0$, we get

$$\begin{aligned} \theta'(t)=\frac{d}{dt}\frac{d}{ds}\overline{z}[x+s\xi ,t]\bigg|_{s=0}, \end{aligned}$$

then we obtain a differential equation for $\theta(t)$

$$\begin{aligned} \theta'(t)-\frac{\partial L[x,z](t)}{\partial z}\theta(t)=\sum _{j=1}^{n} \biggl(\frac{\partial L[x,z](t)}{\partial x_{j}}\xi _{j}(t)+\frac{\partial L[x,z](t)}{\partial{}^{\mathrm {ABC}}_{a}D^{\alpha _{j}}_{t}x_{j}}{}^{\mathrm {ABC}}_{a}D^{\alpha_{j}}_{t} \xi_{j}(t) \biggr), \end{aligned}$$

whose solution is

$$\begin{aligned} \theta(t)\lambda(t)-\theta(a)= \int_{a}^{t}\sum_{j=1}^{n} \biggl(\frac{\partial L[x,z](\tau)}{\partial x_{j}}\xi_{j}(\tau )+\frac{\partial L[x,z](\tau)}{\partial{}^{\mathrm {ABC}}_{a}D^{\alpha _{j}}_{\tau}x_{j}}{}^{\mathrm {ABC}}_{a}D^{\alpha_{j}}_{\tau} \xi_{j}(\tau) \biggr)\lambda(\tau)\,d\tau. \end{aligned}$$

Since $\lambda(t)=\exp (-\int_{a}^{t}\frac{\partial L[x,z](\tau )}{\partial z}\,d\tau )$, $\theta(a)=0$, and for all *τ*, we have $\theta(\tau)=0$. Therefore, we get

$$\begin{aligned} \int_{a}^{t}\sum_{j=1}^{n} \biggl(\frac{\partial L[x,z](\tau)}{\partial x_{j}}\xi_{j}(\tau )+\frac{\partial L[x,z](\tau)}{\partial{}^{\mathrm {ABC}}_{a}D^{\alpha _{j}}_{\tau}x_{j}}{}^{\mathrm {ABC}}_{a}D^{\alpha_{j}}_{\tau} \xi_{j}(\tau) \biggr)\lambda(\tau)\,d\tau=0. \end{aligned}$$

Let $x=(x_{1},x_{2},\ldots,x_{n})$ be such that *z* defined by equation (9) reaches the extremum. According to equation (11), we attain

34 $$\begin{aligned} \lambda(t)\frac{\partial L[x,z](t)}{\partial x_{j}}+{}^{\mathrm {ABR}}_{t}D^{\alpha_{j}}_{b} \biggl(\lambda(t)\frac{\partial L[x,z](t)}{\partial {}^{\mathrm {ABC}}_{a}D^{\alpha_{j}}_{t} x_{j}} \biggr)=0. \end{aligned}$$

Then we get

$$\begin{aligned} \sum^{n}_{j=1} \biggl[\lambda(t) \frac{\partial L[x,z](t)}{\partial{}^{\mathrm {ABC}}_{a}D^{\alpha _{j}}_{t}x_{j}}{}^{\mathrm {ABC}}_{a}D^{\alpha_{j}}_{t}\xi _{j}(t,x)-{}^{\mathrm {ABR}}_{t}D^{\alpha_{j}}_{b} \biggl(\lambda(t)\frac{\partial L[x,z](t)}{\partial {}^{\mathrm {ABC}}_{a}D^{\alpha_{j}}_{t}x_{j}} \biggr)\xi_{j}(t,x) \biggr]=0. \end{aligned}$$

Then we obtain equation (32) with the definition of $\mathcal {D}^{\alpha}[f,g]$. □

## Conclusions

In this paper, fractional calculus of variational Herglotz problem has been discussed with an Atangana–Baleanu fractional derivative. The Euler–Lagrange equations have been received for the Herglotz-type problems, and the necessary optimality conditions, which rely on the reason that the Atangana–Baleanu fractional derivative is non-singular and non-local, have been derived. Fractional variational Herglotz problems of variable order have been considered, and a Noether-type theorem has been proved. However, this paper has only studied a few aspects of fractional calculus. In the future research, we can study what kind of fractional derivatives can be employed in the actual variational problems. As it is difficult to solve the Euler–Lagrange equations, the effective numerical approximate methods should be studied in the future.
